# Mechanisms of neratinib resistance in *HER2*-mutant metastatic breast cancer

**DOI:** 10.20517/cdr.2022.48

**Published:** 2022-09-01

**Authors:** Lisa D. Eli, Shyam M. Kavuri

**Affiliations:** ^1^Translational Medicine and Diagnostics, Puma Biotechnology, Inc., Los Angeles, CA 90024, USA.; ^2^Lester and Sue Smith Breast Center, Baylor College of Medicine, Houston, TX 77030, USA.; ^3^Department of Medicine, Baylor College of Medicine, Houston, TX 77030, USA.

**Keywords:** Neratinib, HER2, *ERBB2*, estrogen receptor, mutation

## Abstract

Human epidermal growth factor receptor 2 (HER2) is a major drug target and clinical biomarker in breast cancer treatment. Targeting *HER2* gene amplification is one of the greatest successes in oncology, resulting in the use of a wide array of HER2-directed agents in the clinic. The discovery of *HER2*-activating mutations as novel therapeutic targets in breast and other cancers marked a significant advance in the field, which led to the metastatic breast and other solid tumor trials MutHER (NCT01670877), SUMMIT (NCT01953926), and one arm of plasmaMATCH (NCT03182634). These trials reported initial clinical benefit followed by eventual relapse ascribed to either primary or acquired resistance. These resistance mechanisms are mediated by additional secondary genomic alterations within *HER2* itself and via hyperactivation of oncogenic signaling within the downstream signaling axis.

## INTRODUCTION

Human epidermal growth factor receptor 2 (HER2)-positive breast cancers have long been treated with targeted therapy, comprising either monoclonal antibodies, such as trastuzumab or pertuzumab, which bind to the extracellular domain of HER2, or tyrosine kinase inhibitors (TKIs), such as the reversible inhibitors lapatinib and tucatinib and the irreversible inhibitor neratinib^[1]^. Genome sequencing efforts have recently identified recurrent somatic mutations in the *HER2 *(*ERBB2*) gene in HER2-negative (non-amplified) breast cancer. Recurrent *HER2* mutations have been proven to be oncogenic drivers in both preclinical experiments and clinical trials^[2-9]^. Activating *HER2* mutations typically fall into four categories, with distribution dependent on tumor type: single nucleotide variants (SNVs) in the extracellular domain, particularly S310F/Y; SNVs in the transmembrane domain; SNVs in the kinase domain; and small insertions in exon 20^[4,10,11]^. *HER2 *mutations constitutively activate the tyrosine kinase receptor activity, leading to upregulation of downstream phosphoinositide 3-kinase (PI3K) and mitogen-activated protein kinase (MAPK) signaling^[6,12]^. *HER2 *mutations are rare in primary cancers, occurring in 2%-12% of solid tumors depending on tumor type and disease stage. In breast cancer, *HER2* mutations vary in frequency from ~2% to 8% depending on disease stage and histology (higher in lobular)^[7,10,11,13,14]^ and have been associated with poor prognosis^[15,16]^. The prevalence of *HER2* mutations is higher in patients with metastatic breast cancer (MBC) that has progressed after primary endocrine therapy (~6%), and these mutations have been causally associated with antiestrogen resistance^[12,13,17]^. Furthermore, *HER2* and estrogen receptor 1 gene (*ESR1*) mutations are mutually exclusive in primary breast cancer, suggesting that *HER2* mutations are independent predictive and prognostic markers in estrogen receptor (ER)-positive MBC^[11,12,16]^.

Neratinib is an orally available, second-generation, pan-HER TKI that irreversibly binds to cysteine residues Cys773 and Cys805 in the ATP pocket of the tyrosine kinase domain of epidermal growth factor receptor (EGFR), HER2, and HER4^[18]^. Neratinib inhibits autophosphorylation, downstream signaling, and growth of EGFR- or HER2-dependent cell lines, with cellular half-maximal inhibitory concentration (IC_50_) < 100 nM^[18]^. Neratinib has been approved by the United States Food and Drug Administration for use in patients with adjuvant and metastatic HER2-positive (overexpressed/amplified) breast cancer based on the results of the ExteNET and NALA trials, respectively^[19,20]^. Furthermore, neratinib has demonstrated significant anti-tumor activity in preclinical models of HER2-negative/non-amplified breast cancer and other solid tumors with *HER2 *mutations^[2,3,5]^. In ER-positive, *HER2-*mutant cell lines, ER signaling was suppressed and cells were resistant to endocrine therapy via estrogen deprivation or fulvestrant treatment; sensitivity was restored upon exposure to neratinib^[17]^. Dual inhibition with neratinib and fulvestrant was required to inhibit the growth of ER-positive, *HER2*-mutant models^[12]^, implying a need to inhibit both the ER and HER2 signaling pathways simultaneously.

Clinically, the utility of neratinib, alone or in combination with other agents, in patients with heavily pretreated *HER2-*mutant breast and other cancers was explored in the phase II SUMMIT and MutHER trials^[4,7]^. The SUMMIT trial demonstrated clinical benefit from single-agent neratinib in patients with several solid tumor types, including *HER2*-mutant breast cancers^[4]^. For patients with *HER2*-mutant, hormone receptor (HR)-positive MBC, both the SUMMIT and MutHER trials were amended to combine neratinib with fulvestrant to suppress both HER2 and HR signaling simultaneously. This dual combination was clinically active in heavily pretreated patients with *HER2*-mutant, HR-positive MBC, including those who had received prior fulvestrant and cyclin-dependent kinase (CDK)4/6 inhibitor therapy. In SUMMIT, the overall response rate (ORR) for neratinib monotherapy in patients with HER2-mutant, HR-positive MBC (*n *= 23) was 17.4%, while the ORR for neratinib plus fulvestrant (*n *= 47) was 29.8%, with clinical benefit rates of 30.4% and 46.8%, respectively^[9]^. Median progression-free survival (PFS) and duration of response (DOR) were also longer with the combination (3.6-month PFS and 6.5-month DOR for neratinib monotherapy; 5.4-month PFS and 9.2-month DOR for neratinib plus fulvestrant)^[9]^. In MutHER, results for neratinib plus fulvestrant (*n *= 31) were clinical benefit rate (CBR) of 30.0%-38.0% and PFS of 5.0-6.0 months^[8,9]^, consistent with the enhanced inhibition observed preclinically^[12,17]^. The independent PlasmaMATCH trial, in which patients with MBC were enrolled based on detection of an activating *HER2* mutation in circulating tumor DNA (ctDNA), reported that neratinib as monotherapy or combined with fulvestrant showed comparable clinical activity when patients were selected using this technique versus when the selection was guided by tissue testing, supporting the utility of ctDNA analysis in this patient population^[21]^.

**Figure 1:**
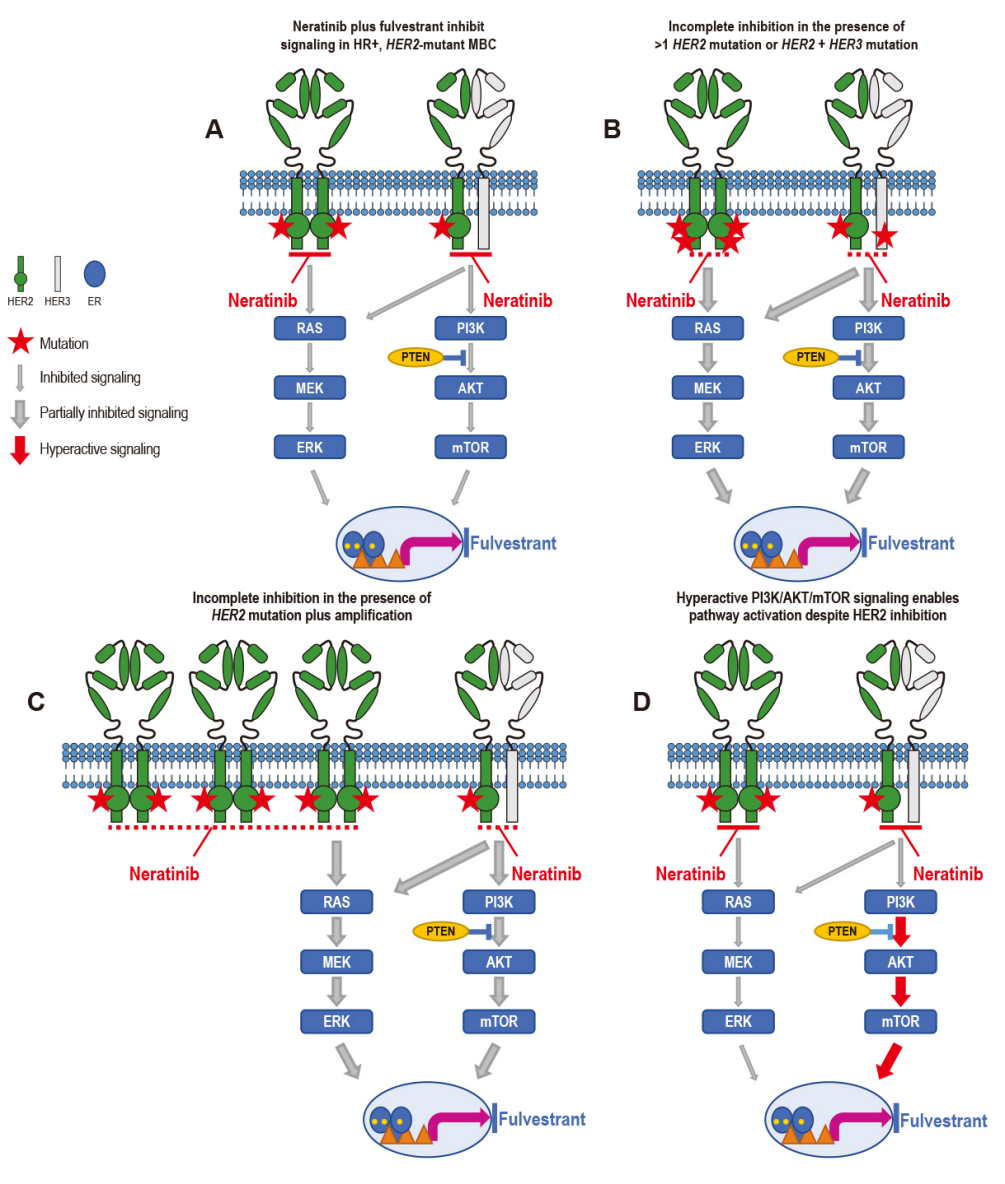
Mechanisms of resistance to neratinib. (A) Neratinib plus fulvestrant inhibit signaling in HR+, *HER*2-mutant MBC. In patients whose tumors harbor a single somatic activating mutation (red star) in *HER2*, neratinib strongly inhibits (thin gray arrow) HER2 pathway signaling, whereas fulvestrant inhibits ER signaling, leading to tumor growth inhibition. (B) Neratinib is less effective against/partially inhibits signaling in (thick gray arrow) tumors with more than one *HER2* mutation or *HER2* mutation plus *HER3* mutation or (C) *HER2* mutation plus amplification, whether these dual alterations are intrinsic or acquired. (D) Hyperactivation (thick red arrow) of downstream signaling can also preclude the effect of neratinib on mutant *HER2*. ER: Estrogen receptor; HR: hormone receptor; MBC: metastatic breast cancer.

Unfortunately, patients who initially derived benefit from neratinib or neratinib plus fulvestrant in these studies eventually relapsed with metastatic disease, and a comparison of the genomic landscape of tumor tissue or ctDNA before treatment and upon progression revealed the acquisition of additional genomic aberrations^[8,9,22]^. Mechanisms of acquired resistance appeared to occur primarily via the development of secondary *HER2* genomic alterations (mutations or amplification), whereas intrinsic resistance was observed not only in patients whose baseline tumors had more than one* HER2* alteration, but also via alterations in the HER3/PI3K/protein kinase B (AKT)/mammalian target of rapamycin (mTOR) and MAPK signaling axis^[8,9,22]^ [Figure 1].

## MECHANISMS OF RESISTANCE

### Accumulation of additional HER2 genomic events

Among patients with heavily pretreated MBC, the presence of more than one *de novo HER2*-activating event before treatment trended with a lack of benefit from neratinib alone or in combination with fulvestrant in trials to date. In the SUMMIT trial, six of the seven patients whose pre-treatment tumors harbored more than one* HER2*-activating event (second *HER2* mutation, *n *= 2; copy-number amplification, *n *= 3; or both, *n *= 2) did not derive clinical benefit^[9]^. In MutHER, three out of 48 patients had dual *HER2* mutations at enrollment; two of those three patients did not experience clinical benefit^[7,8]^.

In patients whose treatment-naive tumors harbored one *HER2 *mutation and who initially derived clinical benefit from neratinib-containing treatments, the one consistently observed mechanism of acquired resistance was the accumulation of a second (or further) additional *HER2* alteration^[8,9]^. In SUMMIT, three of nine patients with HR-positive, *HER2*-mutant MBC, who were treated with neratinib plus fulvestrant and who had both pre- and post-treatment tumors available for central sequencing, had additional *HER2*-activating events in the post-treatment tumor^[9]^. One patient had amplification of the mutant allele, one acquired a gatekeeper mutation, and one had amplification plus two acquired *HER2 *hotspot mutations. Secondary *HER2* mutations were also detected in seven of 16 patients with paired pre-treatment and progression ctDNA who derived clinical benefit, including two of the three described above. Among patients in MutHER who had paired ctDNA samples, acquired *HER2* mutations were detected upon progression in three of six patients with clinical benefit following neratinib monotherapy, in four of seven patients with benefit following neratinib plus fulvestrant, and in one who experienced short-term stable disease^[8]^. Although several of the tumors acquired gatekeeper mutations (T798I and L785F)^[23,24]^, the acquisition of additional sensitizing mutations or variants of unknown significance was also reported [Table 1]. Beyond *HER2*, no other acquired genetic event was consistently observed. These findings suggest that *HER2*-mutant MBCs are dependent on HER2 signaling even upon disease progression.

**Table 1:** *HER2* alterations detected following neratinib-containing regimens in clinical trials of *HER2*-mutant MBC (compiled from the works of Ma *et al*.^[7]^, Ma *et al*.^[8]^, and Smyth *et al*.^[9]^)

**Trial**	**Regimen**	***HER2 *** **mutations detected at baseline**	**Best response**	**Acquired* HER2* alterations**
**MutHER**				
	N	L755S, P761del	PR	R678Q, V697L
	N + F	G778_P780dup	PR	D808H^a^, T798I^b^, I767M
	N + F	S310F	PR	L755S, D769Y, G776V, T798I^b^, L841V
	N + F	G778_P780dup	PR	S310Y, S310F, I767M, T798I^b^
	N	L869R; amplification	SD	D1011D^c^
	N	L869R, D769Y	SD	S310F, I767M, T862A, T798I^b^
	N + F	V777L	SD	S310F
	N + F	L755S	SD*	S310F
**SUMMIT**				
	N + F	S310F	CR	L785F^b^^,^^d^
	N + F	G778_P780dup	PR	I767M, S310Y, amplification^d^
	N + F	L869R	PR	S310Y, D769Y, L755S, T798I^b^
	N + F	V697L	PR	Amplified mutant allele^d^
	N + F	V777L	PR	T798I^b^
	N + F	L755S, L755P	SD	T862A, S310F
	N + F	G776V	SD	I767M
	N + F	L755S	PD*	D769H, D962H^a^, K1171N^a^, D1016Y^a^, D1089Y^a^

All data are from circulating tumor DNA sequencing performed by Guardant360 for both MutHER and SUMMIT trials unless noted otherwise. All patients except those marked with an asterisk (*) achieved clinical benefit. ^a^Variant of unknown significance. ^b^Gatekeeper mutation. ^c^Synonymous mutation. ^d^Tissue samples, sequenced by Memorial Sloan Kettering-Integrated Mutation Profiling of Actionable Cancer Targets (MSK-IMPACT). CR: Complete response; F: fulvestrant; MBC: metastatic breast cancer; N: neratinib; PD: progressive disease; PR: partial response; SD: stable disease.

### Aberrant HER3/PI3K/mTOR signaling

In preclinical models of HR-positive breast cancer, the recurrent *HER2* L755S and V777L mutations constitutively upregulated HER3 phosphorylation, particularly upon treatment with fulvestrant, resulting in hyperactivation of the HER3/PI3K/AKT/mTOR signaling axis and leading to antiestrogen resistance^[12,22]^. Structural modeling of the *HER2* L755S mutation revealed a loss of flexibility in the active state, allowing for increased HER2/HER3 heterodimerization and upregulation of PI3K/AKT/mTOR signaling^[12]^. *HER3* mutations have been modeled to stabilize HER2/HER3 dimerization and increase HER2 signaling^[25,26]^, and preclinical models showed that dual *HER2/HER3* mutations further enhanced oncogenicity and promoted resistance to HER2-targeted therapies, including neratinib^[26]^. In SUMMIT, pre-existing concurrent activating *HER3 *mutations were associated with poor treatment outcomes in patients with *HER2*-mutant MBC^[9]^. Further analysis of data from SUMMIT patients with *HER2*-mutant tumors across multiple tumor types revealed that mTOR pathway alterations were associated with a lack of clinical benefit with single-agent neratinib. Preclinically, hyperactivation of mTOR signaling was an actionable acquired mechanism of resistance to neratinib in *HER2-*mutant cell lines and patient-derived xenograft (PDX) models^[22]^. Interestingly, however, *PIK3CA* mutations *per se* were not associated with a lack of clinical benefit with neratinib plus fulvestrant in *HER2*-mutant MBC^[8,9]^, and no other single gene or mutation in the HER3/PI3K/AKT/mTOR signaling axis was clearly associated with acquired neratinib resistance.

### Acquisition of somatic *HER2* mutations in HER2-positive breast cancer

Although this review focuses specifically on the acquisition of resistance to neratinib in HER2-negative, *HER2*-mutant MBC, the acquisition of *HER2 *mutations in HER2-positive breast cancer also merits consideration. The co-occurrence of *HER2* mutations and amplification has been associated with poor response to trastuzumab and lapatinib, although neratinib has been shown to be effective against HER2-positive, *HER2*-mutant preclinical models and in patients whose breast tumors had coincident *HER2* amplification and mutation, suggesting neratinib as monotherapy may be effective in this setting^[27]^. In preclinical models of HER2-positive breast cancer, HER2 reactivation in lapatinib-resistant derivatives was driven by the acquisition of a *HER2 *L755S mutation, which could be overcome by neratinib or afatinib^[28]^. Finally, recent findings from plasmaMATCH demonstrate that the incidence of* HER2* mutations in HER2-positive cancers increases with the number of lines of HER2-directed therapy^[29]^. Regardless of initial HER2-positive or *HER2*-mutant status, accumulation of genomic events within *HER2 *itself is prevalent upon exposure to HER2-targeted agents. Whether or not there is a clinical difference in response depending on which type of alteration is the initial driver remains to be seen.

Taken together, these findings provide a rationale for the combination of multiple HER2 inhibitors or inhibitors of the downstream signaling axis in patients with *HER2*-mutant breast cancer, a therapeutic strategy that has already proven highly effective in HER2-positive breast cancer.

## OVERCOMING NERATINIB RESISTANCE IN *HER2*–MUTANT MBC

The following are three possible approaches to overcoming neratinib resistance in *HER2*-mutant MBC: (1) dual HER2 targeting; (2) combination with PI3K, mTOR, MEK, or CDK4/6 inhibitors; and (3) sequential TKI treatment.

### Dual HER2 targeting: neratinib plus monoclonal antibody or antibody–drug conjugate

The dual targeting of HER2 either upfront or at disease progression has proven to be effective in patients with *HER2*-mutant MBC. In MutHER, adding trastuzumab after disease progression on neratinib plus fulvestrant led to re-response in four of five patients, with a concomitant decrease in ctDNA^[8]^. In SUMMIT, the *HER2*-mutant breast cohorts were recently amended to treat patients upfront with the triple combination of neratinib, fulvestrant, and trastuzumab. This combination, in fact, demonstrated encouraging clinical activity in SUMMIT patients with heavily pretreated HR-positive, HER2-negative, *HER2*-mutant MBC who had previously received a CDK4/6 inhibitor (*n *= 33; ORR of 42.4%, CBR of 51.5%, median DOR of 14.4 months, median PFS of 7.0 months)^[30,31]^. Preclinically, neratinib combined with trastuzumab in *HER2*-mutant cancer models yielded more robust inhibition of HER2 signaling and growth than either agent alone^[5,32]^.

Neratinib induces HER2 receptor ubiquitination and endocytosis^[33]^; combining neratinib with a HER2-directed antibody–drug conjugate may therefore enable increased payload internalization. In *HER2*-mutant PDX models, the combination of neratinib and trastuzumab emtansine (T-DM1) or trastuzumab deruxtecan (T-DXd) did, in fact, show synergistic tumor growth inhibition^[34]^. Safety and preliminary efficacy of neratinib plus T-DM1 have been demonstrated in patients with HER2-positive breast cancer^[35]^; clinical trials of neratinib plus antibody–drug conjugates are similarly warranted in the *HER2*-mutant MBC setting.

### Combination with PI3K, mTOR, MEK, or CDK4/6 inhibitors

Combining neratinib with inhibitors of the downstream signaling axis or with CDK4/6 inhibitors may be a second approach to prolonging response to neratinib in patients with *HER2*-mutant MBC. First, preclinical data in *HER2/HER3* double mutant cell lines show that the combination of a PI3K inhibitor (alpelisib) with neratinib overcame neratinib resistance^[26]^. Second, the combination of the mTOR inhibitor everolimus with neratinib arrested the growth of neratinib-resistant, ER-positive, *HER2*-mutant organoids and xenografts^[22]^. Third, in two HER2-positive breast and colorectal PDX models harboring activating *HER2* mutations (V777L and R678Q) derived from patients who had been treated with HER2-targeted therapies, the combination of neratinib with the MEK inhibitor trametinib, the mTOR inhibitors everolimus or sapanisertib, or the CDK4/6 inhibitor palbociclib synergistically decreased tumor volume to a greater extent than any of the agents alone. These combinations were well tolerated in HER2-positive preclinical PDX models^[36]^. A clinical trial to study the safety and tolerability of neratinib combined with trametinib, everolimus, or palbociclib in metastatic solid tumors with HER family alteration or *KRAS* mutation is currently underway (NCT03065387)^[37]^. Given the promising efficacy of neratinib-containing regimens post CDK4/6 inhibitor in the SUMMIT trial^[31]^, first-line treatment with neratinib plus a CDK4/6 inhibitor and endocrine therapy could warrant investigation if the combination is deemed tolerable.

### Sequential treatment of neratinib with a second TKI

Sequential TKI treatment has long been standard in *EGFR*-mutant non-small cell lung cancer, and a similar approach could be investigated for *HER2*-mutant MBC. The *HER2* gatekeeper mutation T798I is recurrent in *HER2*-mutant MBC upon clinical progression following neratinib. Preclinically, another second-generation TKI, afatinib, and AZ5104, the metabolite of the third-generation TKI osimertinib, blocked *HER2* T798I mutation-induced cell growth and signaling^[23]^. These findings support the clinical investigation of sequencing TKI therapy in *HER2-*mutant cancers that develop gatekeeper mutations.

## CONCLUSION

*HER2*-activating mutations are a targetable alteration in MBC and can be inhibited by neratinib. In heavily pretreated patients with MBC, more than one alteration in the HER2 signaling pathway, whether in the *HER2* gene itself or downstream in the signaling cascade, may preclude initial response. Furthermore, patients with a single *HER2* mutation who derive initial clinical benefit appear to become resistant via the acquisition of additional *HER2 *mutations and/or amplification. Dual HER2-targeting via the addition of trastuzumab to neratinib, in combination with fulvestrant for patients with HR-positive MBC, has exhibited strong clinical activity against *HER2-*mutant MBC^[8,30,31]^; targeting both HER2 and CDK4/6 together may warrant exploration as part of a front-line approach in this setting. Future analysis of plasma samples from patients receiving dual HER2-targeting will elucidate whether acquired resistance occurs via the same mechanisms. Combination and/or sequencing of neratinib plus additional agents targeting either HER2 or downstream or alternative pathway members may be required for more durable clinical benefit. Any combination approach will require diligent clinical management given the gastrointestinal toxicity profile of neratinib, although neratinib dose escalation may help to mitigate adverse events^[38]^.

Future studies may consider molecularly guided approaches beyond genomics, including but not limited to evaluation of changes in gene or protein expression or protein phosphorylation status, to inform the design of rational drug combinations and lead to improved outcomes for patients with *HER2*-mutant MBC.

## DECLARATIONS

### Copyright

© The Author(s) 2022.

## References

[1] Kunte S, Abraham J, Montero AJ (2020). Novel HER2-targeted therapies for HER2-positive metastatic breast cancer. Cancer.

[2] Bose R, Kavuri SM, Searleman AC (2013). Activating HER2 mutations in HER2 gene amplification negative breast cancer. Cancer Discov.

[3] Shimamura T, Ji H, Minami Y (2006). Non-small-cell lung cancer and Ba/F3 transformed cells harboring the ERBB2 G776insV_G/C mutation are sensitive to the dual-specific epidermal growth factor receptor and ERBB2 inhibitor HKI-272. Cancer Res.

[4] Hyman DM, Piha-Paul SA, Won H (2018). HER kinase inhibition in patients with HER2- and HER3-mutant cancers. Nature.

[5] Kavuri SM, Jain N, Galimi F (2015). HER2 activating mutations are targets for colorectal cancer treatment. Cancer Discov.

[6] Zabransky DJ, Yankaskas CL, Cochran RL (2015). HER2 missense mutations have distinct effects on oncogenic signaling and migration. Proc Natl Acad Sci USA.

[7] Ma CX, Bose R, Gao F (2017). Neratinib efficacy and circulating tumor DNA detection of HER2 mutations in HER2 nonamplified metastatic breast cancer. Clin Cancer Res.

[8] Ma CX, Luo J, Freedman RA (2022). The phase II MutHER study of neratinib alone and in combination with fulvestrant in HER2-mutated, non-amplified metastatic breast cancer. Clin Cancer Res.

[9] Smyth LM, Piha-Paul SA, Won HH (2020). Efficacy and determinants of response to HER kinase inhibition in HER2-mutant metastatic breast cancer. Cancer Discov.

[10] Schram A, Won HH, Andre F (2017). Abstract LB-103: landscape of somatic ERBB2 mutations: findings from AACR GENIE and comparison to ongoing ERBB2 mutant basket study. Cancer Res.

[11] Connell CM, Doherty GJ (2017). Activating HER2 mutations as emerging targets in multiple solid cancers. ESMO Open.

[12] Croessmann S, Formisano L, Kinch LN (2019). Combined blockade of activating ERBB2 mutations and ER results in synthetic lethality of ER+/HER2 mutant breast cancer. Clin Cancer Res.

[13] Razavi P, Chang MT, Xu G (2018). The genomic landscape of endocrine-resistant advanced breast cancers. Cancer Cell.

[14] Desmedt C, Zoppoli G, Gundem G (2016). Genomic characterization of primary invasive lobular breast cancer. J Clin Oncol.

[15] Wang T, Xu Y, Sheng S (2017). HER2 somatic mutations are associated with poor survival in HER2-negative breast cancers. Cancer Sci.

[16] Kurozumi S, Alsaleem M, Monteiro CJ (2020). Targetable ERBB2 mutation status is an independent marker of adverse prognosis in estrogen receptor positive, ERBB2 non-amplified primary lobular breast carcinoma: a retrospective in silico analysis of public datasets. Breast Cancer Res.

[17] Nayar U, Cohen O, Kapstad C (2019). Acquired HER2 mutations in ER^+^ metastatic breast cancer confer resistance to estrogen receptor-directed therapies. Nat Genet.

[18] Rabindran SK, Discafani CM, Rosfjord EC (2004). Antitumor activity of HKI-272, an orally active, irreversible inhibitor of the HER-2 tyrosine kinase. Cancer Res.

[19] Martin M, Holmes FA, Ejlertsen B (2017). Neratinib after trastuzumab-based adjuvant therapy in HER2-positive breast cancer (ExteNET): 5-year analysis of a randomised, double-blind, placebo-controlled, phase 3 trial. Lancet Oncol.

[20] Saura C, Oliveira M, Feng YH, NALA Investigators (2020). Neratinib plus capecitabine versus lapatinib plus capecitabine in HER2-positive metastatic breast cancer previously treated with ≥ 2 HER2-directed regimens: phase III NALA trial. J Clin Oncol.

[21] Turner NC, Kingston B, Kilburn LS (2020). Circulating tumour DNA analysis to direct therapy in advanced breast cancer (plasmaMATCH): a multicentre, multicohort, phase 2a, platform trial. Lancet Oncol.

[22] Sudhan DR, Guerrero-Zotano A, Won H (2020). Hyperactivation of TORC1 drives resistance to the pan-HER tyrosine kinase inhibitor neratinib in HER2-mutant cancers. Cancer Cell.

[23] Hanker AB, Brewer MR, Sheehan JH (2017). An acquired HER2^T798I^ gatekeeper mutation induces resistance to neratinib in a patient with HER2 mutant-driven breast cancer. Cancer Discov.

[24] Smyth L, Piha-Paul S, Saura C (2019). Abstract PD3-06: neratinib + fulvestrant for HER2-mutant, HR-positive, metastatic breast cancer: updated results from the phase 2 SUMMIT trial. Cancer Res.

[25] Collier TS, Diraviyam K, Monsey J, Shen W, Sept D, Bose R (2013). Carboxyl group footprinting mass spectrometry and molecular dynamics identify key interactions in the HER2-HER3 receptor tyrosine kinase interface. J Biol Chem.

[26] Hanker AB, Brown BP, Meiler J (2021). Co-occurring gain-of-function mutations in HER2 and HER3 modulate HER2/HER3 activation, oncogenesis, and HER2 inhibitor sensitivity. Cancer Cell.

[27] Cocco E, Javier Carmona F, Razavi P (2018). Neratinib is effective in breast tumors bearing both amplification and mutation of ERBB2 (HER2). Sci Signal.

[28] Xu X, De Angelis C, Burke KA (2017). HER2 reactivation through acquisition of the HER2 L755S mutation as a mechanism of acquired resistance to HER2-targeted therapy in HER2^+^ breast cancer. Clin Cancer Res.

[29] Kingston B, Cutts RJ, Bye H (2021). Genomic profile of advanced breast cancer in circulating tumour DNA. Nat Commun.

[30] Jhaveri K, Saura C, Guerrero-Zotano A (2021). Abstract PD1-05: latest findings from the breast cancer cohort in SUMMIT - a phase 2 ‘basket’ trial of neratinib + trastuzumab + fulvestrant for HER2 -mutant, hormone receptor-positive, metastatic breast cancer. Cancer Res.

[31] Jhaveri K, Park H, Waisman J (2022). Abstract GS4-10: neratinib + fulvestrant + trastuzumab for hormone receptor-positive, HER2-mutant metastatic breast cancer and neratinib + trastuzumab for triple-negative disease: Latest updates from the SUMMIT trial. Cancer Res.

[32] Ivanova E, Kuraguchi M, Xu M (2020). Use of ex vivo patient-derived tumor organotypic spheroids to identify combination therapies for HER2 mutant non-small cell lung cancer. Clin Cancer Res.

[33] Zhang Y, Zhang J, Liu C (2016). Neratinib induces ErbB2 ubiquitylation and endocytic degradation via HSP90 dissociation in breast cancer cells. Cancer Lett.

[34] Bose R, Li S, Primeau TM (2021). Abstract PS4-13: irreversible inhibition of HER2 activating mutations with neratinib enhances the pre-clinical efficacy of trastuzumab emtansine and trastuzumab deruxtecan. Cancer Res.

[35] Abraham J, Montero AJ, Jankowitz RC (2019). Safety and efficacy of T-DM1 plus neratinib in patients with metastatic HER2-positive breast cancer: NSABP foundation trial FB-10. J Clin Oncol.

[36] Zhao M, Scott S, Evans KW (2021). Combining neratinib with CDK4/6, mTOR, and MEK inhibitors in models of HER2-positive cancer. Clin Cancer Res.

[37] Piha-Paul SA, Fu S, Hong DS (2018). Phase I study of the pan-HER inhibitor neratinib given in combination with everolimus, palbociclib or trametinib in advanced cancer subjects with EGFR mutation/amplification, HER2 mutation/amplification or HER3/4 mutation. J Clin Oncol.

[38] Chan A, Ruiz-Borrego M, Marx G (2022). Abstract P5-18-02: final findings from the CONTROL trial of diarrheal prophylaxis or neratinib dose escalation on neratinib-associated diarrhea and tolerability in patients with HER2+ early-stage breast cancer. Cancer Res.

